# Error Estimation for the Linearized Auto-Localization Algorithm

**DOI:** 10.3390/s120302561

**Published:** 2012-02-24

**Authors:** Jorge Guevara, Antonio R. Jiménez, Jose Carlos Prieto, Fernando Seco

**Affiliations:** Centro de Automática y Robótica (CAR), Consejo Superior de Investigaciones Científicas (CSIC)-UPM, Ctra. Campo Real km 0.2, La Poveda-Arganda del Rey, 28500, Madrid, Spain; E-Mails: antonio.jimenez@csic.es (A.R.J.); josecarlos.prieto@car.upm-csic.es (J.C.P.); fernando.seco@car.upm-csic.es (F.S.)

**Keywords:** auto-localization, auto-calibration, local positioning systems, differential sensitivity analysis, uncertainty propagation

## Abstract

The Linearized Auto-Localization (LAL) algorithm estimates the position of beacon nodes in Local Positioning Systems (LPSs), using only the distance measurements to a mobile node whose position is also unknown. The LAL algorithm calculates the inter-beacon distances, used for the estimation of the beacons’ positions, from the linearized trilateration equations. In this paper we propose a method to estimate the propagation of the errors of the inter-beacon distances obtained with the LAL algorithm, based on a first order Taylor approximation of the equations. Since the method depends on such approximation, a confidence parameter *τ* is defined to measure the reliability of the estimated error. Field evaluations showed that by applying this information to an improved weighted-based auto-localization algorithm (WLAL), the standard deviation of the inter-beacon distances can be improved by more than 30% on average with respect to the original LAL method.

## 1. Introduction

There are many location-aware applications that require the estimation of the position of persons or objects in indoor environments. Since the Global Positioning System (GPS) is not available inside buildings, several localization systems have been designed to work in indoor environments, which are commonly known as Local Positioning Systems (LPSs) [1]. These systems require the installation of several nodes at fixed positions (called beacons) in the indoor environment, whose positions must be known in advance. The location of a mobile node (attached to the person or object to locate) can be obtained by the trilateration method using the measured distances between the beacons and the mobile node.

The manual determination of the beacons’ positions (e.g., using measuring tapes or ultrasonic/laser rangers) is a cumbersome error-prone method. Therefore various techniques have been proposed to address the problem of obtaining the position of the beacons [2–11], sometimes called the auto-calibration or auto-localization problem.

Typical auto-localization solutions are based on measuring distances from the beacons to a group of localized nodes, or a mobile node at several known positions, and performing an inverse trilateration (locating the beacons using the mobile node) [2]. In [4] four different mobile node positions are used to locate the beacons of a 3D LPS. In [3] three nodes with known positions are required plus a group of nodes with unknown positions. In [5] only the relative distances between four nodes mounted on a mobile robot are required. These methods, however, require an external localization system to obtain the position of the mobile node at each static location, which is not always available in indoor environments.

Assuming that no information on the position of any node is known, the only available data are the distance measurements between the beacons and the mobile node. Using this information Duff and Muller [6] proposed a nonlinear least-square optimization algorithm, where the objective functions were the distance equations and the variables the coordinates of all nodes. The initial conditions for the algorithm (a first position estimation of all nodes) were obtained by a trial and error method, by randomly generating those conditions and choosing the best solution. In [7] and [8] an Extended Kalman Filter and an H-infinite filter are used, respectively, to obtain the position of the beacons and the mobile node. In both cases an initial position estimation for the beacons is obtained using dead-reckoning information of the mobile node. In [9], a distance matrix is formed with the range measurements between beacons and the mobile node at different locations. With that information a rough approximation of the inter-beacon ranges is obtained using an interpolation scheme.

In [10] a solution of the auto-localization problem, based on the linearization of the trilateration equations, was presented. The method, known as the Linearized Auto-Localization (LAL) algorithm, neither requires a trial and error approximation (*i.e.*, randomly generated positions) nor any external positioning information (such as dead-reckoning data). The LAL algorithm is used when all beacons are located in a plane (e.g., when all the beacons are located on the ceiling) parallel to the plane containing the mobile node trajectory. By using these conditions, all nonlinear terms of the trilateration equations can be grouped in additional variables in order to obtain linear auto-localization equations.

In the present paper we propose a method to estimate the solution’s standard deviation of the LAL algorithm based on a first order Taylor approximation. Since the method depends on the assumption that the solution has a Gaussian distribution, a confidence parameter *τ* is defined to measure the reliability of the estimated standard deviation. The information obtained from the solution’s precision is later used to further improve the LAL algorithm.

This paper is organized as follows. Section 2 describes the LAL algorithm [10]. In Section 3 the equations to estimate the solution’s standard deviation and their reliability are proposed. These are evaluated by simulation in Section 4 and also experimentally on an ultrasonic 3D LPS system in Section 6. Section 7 presents our conclusions.

## 2. The LAL Algorithm

**Figure 1.**
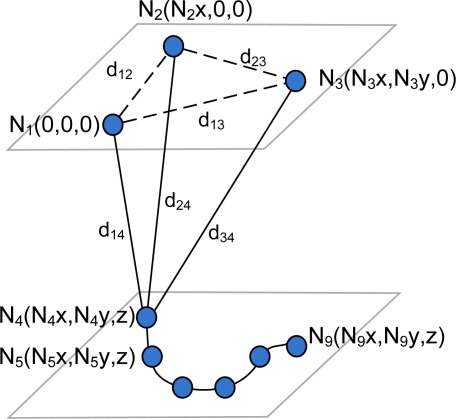
Solvable node subset composed by three beacon nodes and six virtual nodes on a plane.

In [10] a new solution for the auto-localization problem of 3D LPSs was presented based on the distance measurements between beacons and the mobile node. While other techniques require an external positioning system (e.g., a second LPS or an odometric sensor) to estimate the positions of the mobile node, the proposed method uses only the measurements available to the LPS being used for mobile node location. The method is based on the linearization of the trilateration equations by grouping nonlinear terms in additional variables. The LAL algorithm is defined for the case where all the beacons are located in a plane parallel to the plane containing the mobile node trajectory. For this particular configuration, a solvable initial subset of three beacon nodes is obtained based on [11]. Figure 1 shows the auto-localization configuration for *n* = 3 beacon nodes *N_i_*, *i* = {1, 2, 3} and *m* measurement points *N_j_*, *j* = {4, . . ., *m* + 3} from the mobile node path. For *n* = 3 beacons nodes a minimum of *m* = 6 measurements points are required [11], though more measurement points can be added to improve the estimated solution. From now on, the measurement points of the mobile node will be referred to as *virtual nodes*.

For a configuration of *n* = 3 beacons and *m* = 6 virtual nodes, the trilateration equations can be written as a function of two groups of distances: the unknown inter-beacon distances *D_U_* = {*d*_12_, *d*_13_, *d*_23_} (the variables to be estimated) and the measured distances *D_M_* = {*d*_14_, *d*_24_, *d*_34_, . . ., *d*_39_} between beacon nodes and virtual nodes (the available data). The final equation is expressed in the linear form [10]:

(1) AX=B

where:

(2) $$A=\left[\begin{array}{ccccc} d_{34}^{2}-d_{39}^{2} & d_{24}^{2}-d_{29}^{2} & d_{14}^{2}-d_{19}^{2} & E_{324}E_{214}-E_{329}E_{219} & -E_{314}E_{214}+E_{319}E_{219}\\ d_{35}^{2}-d_{39}^{2} & d_{25}^{2}-d_{29}^{2} & d_{15}^{2}-d_{19}^{2} & E_{325}E_{215}-E_{329}E_{219} & -E_{315}E_{215}+E_{319}E_{219}\\ \vdots & \vdots & \vdots & \vdots & \vdots \\ d_{38}^{2}-d_{39}^{2} & d_{28}^{2}-d_{29}^{2} & d_{18}^{2}-d_{19}^{2} & E_{328}E_{218}-E_{329}E_{219} & -E_{318}E_{218}+E_{319}E_{219} \end{array}\right]$$

(3) $$E_{\mathrm{ikj}}=d_{\mathrm{ij}}^{2}-d_{\mathrm{kj}}^{2},\;\;i,k=\{1,2,3\}\;\;j=\{4,5,\ldots ,9\}$$

(4) $$X=\frac{1}{d_{12}^{2}}\;\left[\begin{array}{c} d_{12}^{2}(d_{13}^{2}+d_{23}^{2}-d_{12}^{2})\\ d_{13}^{2}(d_{12}^{2}+d_{23}^{2}-d_{13}^{2})\\ d_{23}^{2}(d_{12}^{2}+d_{13}^{2}-d_{23}^{2})\\ d_{13}^{2}\\ d_{23}^{2} \end{array}\right]$$

(5) $$B=\left[\begin{array}{c} E_{314}\;E_{324}-E_{319}\;E_{329}\\ E_{315}\;E_{325}-E_{319}\;E_{329}\\ \vdots \\ E_{318}\;E_{328}-E_{319}\;E_{329} \end{array}\right]$$

Matrix *A* ∈ ℝ^(*m*−1)×5^ and vector *B* ∈ ℝ^(*m*−1)×1^ are composed of the distance measurements from the virtual nodes *D^M^*. The vector *X* ∈ ℝ^5×1^ includes the unknown inter-beacon distances. Once *X* is obtained the inter-beacon distances solution *D^U^* can be calculated with:

(6) $$\begin{array}{l} d_{12}=\frac{1}{\sqrt{2}}\;\sqrt{\frac{X_{2}}{X_{4}}+\frac{X_{3}}{X_{5}}}\\ d_{13}=\frac{1}{\sqrt{2}}\;\sqrt{X_{1}+\frac{X_{3}}{X_{5}}}\\ d_{23}=\frac{1}{\sqrt{2}}\;\sqrt{X_{1}+\frac{X_{2}}{X_{4}}} \end{array}$$

For more than three beacon nodes an incremental procedure is used. The mobile node is moved on a plane trying to obtain at least six measurements shared by subsets of three beacon nodes. The goal is to obtain all possible inter-beacon solutions *D^U^* applying the linearized Equation (1) on such subsets. In large areas, or if the range of the nodes is limited, multiple paths can be required. Once enough inter-beacon distances have been obtained, a 2D localization algorithm is used to locate every beacon position. In the LAL method an algorithm based on the Multidimensional Scaling (MDS) is used to obtain all the beacons’ positions [12]. The MDS algorithm requires the distance matrix which represents the pairwise distances between all the beacons. Since for large localization areas it is difficult to move the mobile node in a path where all beacons are in range, some inter-beacon distances must be estimated by other means like the nearest path between those beacons. However, this estimation is a rough approximation of the real inter-beacons distances and it has a negative impact in the precision of the MDS method. To avoid this problem, the LAL algorithm uses a modification of the MDS proposed in [13] known as LaMSM. This method locates only subsets of fully connected beacons forming local maps that are later merged in a global map containing all beacons.

## 3. Precision of the Auto-Localization Method

Consider that the distance measurements *D^M^*, between beacons and virtual nodes, are given by:

(7) $$d_{\mathrm{ij}}=\bar{d_{\mathrm{ij}}}+{ɛ}_{\mathrm{ij}}$$

where $\bar{d_{\mathrm{ij}}}$ is the true distance between beacon *i* = {1, 2, 3} and virtual node *j* = {4, . . ., *m* + 3}, and *ɛ_ij_* is a zero mean Gaussian distribution with variance $\sigma_{\mathrm{ij}}^{2}$. An estimation of the precision of the inter-beacon distances *D^U^* obtained by the LAL algorithm presented in Section 2 would be very useful in the posterior determination of the beacons’ location. However, since it is clear that the resultant error distribution of *D^U^* is not Gaussian, a direct evaluation of such error can be a complex task. A good estimation can be obtained by using a first order approximation of the nonlinear equations based on a technique called differential sensitivity analysis [14].

### 3.1. Differential Sensitivity Analysis

Differential Sensitivity Analysis (DSA) is a technique used to evaluate the error of a given function originated by the uncertainty present on its variables. This technique uses a first order Taylor expansion to obtain an approximation of the function’s variance.

Consider *l* variables *y_u_* = *g_u_*(*Z*), *u* = {1, .., *l*}, each one function of *p* uncorrelated variables *Z* = {*z*_1_, . . ., *z_p_*} normally distributed with mean *z̄_k_* and variance $\sigma_{z_{k}}^{2}$. The variance *V* (*y_u_*) of each variable *y_u_* can be expressed as:

(8) $$V(y_{u})=\sum_{k=1}^{p}{\left(\frac{\partial g_{u}}{\partial z_{k}}\right)}^{2}\;\sigma_{z_{k}}^{2}$$

and the covariance *C*(*y_u_*, *y_v_*) between variables *y_u_* and *y_v_*:

(9) $$C(y_{u},\;y_{v})=\sum_{k=1}^{p}\left(\frac{\partial g_{u}}{\partial z_{k}}\right)\;\;\left(\frac{\partial g_{v}}{\partial z_{k}}\right)\;\;\sigma_{z_{k}}^{2}$$

Equations (8) and (9) can be written in matrix form to obtain the covariance matrix *C*(*Y*) of vector *Y* = [*y*_1_, . . ., *y_l_*]*^T^* [15]:

(10) $$C(Y)=𝒢C(Z){𝒢}^{T}$$

where *C*(*Z*) is the covariance matrix of *Z* and 𝒢 is the Jacobian matrix of *G* = {*g*_1_, . . ., *g_l_*} with respect to vector *Z*.

The approximation obtained with the DSA is only valid if the real distribution of *Y* is close to a Gaussian distribution, that is, if the higher terms of the Taylor series can be neglected. It is important to verify the range of application of the method, otherwise the estimated parameters could be significantly different from the true ones. The range of validity of this approximation will be further discussed in the present section.

In the next subsection we will apply the DSA to obtain the variance of the least squares solution of Equation (1).

### 3.2. Error Perturbation in Least Squares Solutions

For the estimation of the error in the least squares solution, we will use an approximation proposed by Stewart [16]. Similarly to the differential technique previously used, it is based on the assumption that the error can be modelled as a Gaussian distribution. Although in [16] this approximation was obtained for the case when the components of matrix *A* and vector *B* are row-wise independent, we extend it to a more general case where all elements of *A* and *B* can be dependent.

Let us define the observed matrices *A*′ = *A*+ *Ã* and *B*′ = *B* + *B̃*, where *Ã* and *B̃* represent the error perturbation on the true matrices *A* and *B*, originated by the noisy distance measurements. Matrices *A*′ and *B*′ are the ones used to obtain the least squares solution, since we do not know the actual values *A* and *B* of such matrices. Equation (1) can be rewritten as:

(11) $$(A^{\prime }-\tilde{A})X=B^{\prime }-\tilde{B}$$

left multiplying by the pseudo-inverse *A*′^+^ = (*A′^T^ A*′)^−1^ *A*′*^T^* and rearranging the equation we obtain:

(12) $$X^{\prime }=X+{A^{\prime }}^{+}(\tilde{B}-\tilde{A}X)$$

where *X*′ = *A*′^+^ *B*′ is the calculated solution we want to evaluate. The pseudo-inverse *A*′^+^ can be expressed as a series function of matrix *A* [16]:

(13) $$X^{\prime }=X+A^{+}(\tilde{B}-\tilde{A}X)+F(\tilde{B}-\tilde{A}X)$$

where *F* represents the higher order terms of *A*′^+^.

Finally we can simplify Equation (13) to obtain the approximation:

(14) $$X^{\prime }=X+A^{+}(\tilde{B}-\tilde{A}X)$$

where the step from Equation (13) to Equation (14) is based on the hypothesis that *A*^+^ ≫ *F*. Notice that Equation (14) is not a first order approximation of Equation (13), but rather a more restricted one.

Using Equation (14) we can obtain the desired covariance matrix *C*(*X*′) of the least squares solution *X*′, which is given by:

(15) $$\begin{array}{ll} C(X^{\prime }) & =C(A^{+}(\tilde{B}-\tilde{A}X))\\ & =A^{+}C(\tilde{B}-\tilde{A}X)A^{+T}\\ & =A^{+}ℛC(D^{M}){ℛ}^{T}A^{+T} \end{array}$$

where the ℛ is the Jacobian matrix of *R* = *B* − *AX* with respect to *D^M^*.

### 3.3. Covariance Matrix of the Inter-Beacon Distances

The covariance matrix *C*(*D^U^*) of the inter-beacon distances *D^U^* is obtained by applying Equation (10) to the distance Equation (6):

(16) $$C(D^{U})=ℋC(X^{\prime }){ℋ}^{T}$$

where ℋ represents the Jacobian matrix of Equation (6) with respect to *X*.

Replacing Equation (15) in Equation (16) we obtain the final variance error of the LAL method:

(17) $$C(D^{U})=ℋA^{+}ℛC(D^{M}){ℛ}^{T}A^{+T}{ℋ}^{T}$$

Considering the simplest case, where all the measured distances *D^M^* have the same variance $\sigma_{\mathrm{ij}}^{2}=\sigma_{M}^{2}$, *i* = {1, 2, 3} *j* = {4, . . ., *m* + 3} we have that Equation (17) can be simplified as:

(18) $$\begin{array}{c} \begin{array}{ll} C(D^{U}) & =\sigma_{M}^{2}ℋA^{+}ℛ{ℛ}^{T}A^{+T}{ℋ}^{T}\\ & =\sigma_{M}^{2}\;M_{\mathrm{DOP}} \end{array} \end{array}$$

where the effect of matrix *M_DOP_* ∈ ℝ^3×3^ is equivalent to the effect of the geometric dilution of precision (GDOP) in GPS [17]. For our case, we define the Distance Dilution of Precision (DDOP) as the amplification of the standard deviation *σ_M_* of the measurement errors*D^M^* onto the inter-beacon distances *D^U^*. This amplification only depends on the relative position of the nodes (beacons and virtuals).

For each inter-beacon distance *d_ik_* *i*, *k* = {1, 2, 3}, between beacons *i* and *k*, a respective distance dilution of precision *ddop_ik_* can be obtained by:

(19) $$\left[\begin{array}{c} {\mathrm{ddop}}_{12}\\ {\mathrm{ddop}}_{13}\\ {\mathrm{ddop}}_{23} \end{array}\right]=\frac{1}{\sigma_{M}}\;\left[\begin{array}{c} \sigma_{12}\\ \sigma_{13}\\ \sigma_{23} \end{array}\right]=\left[\begin{array}{c} \sqrt{m_{{\mathrm{DOP}}_{11}}}\\ \sqrt{m_{{\mathrm{DOP}}_{22}}}\\ \sqrt{m_{{\mathrm{DOP}}_{33}}} \end{array}\right]$$

where *σ_ik_* is the standard deviation of the distance *d_ik_* and *m_DOP_uv__* denotes the (*uv*) element of matrix *M_DOP_* .

### 3.4. Reliability of the Variance Estimation

As discussed in Section 3.1, the variance estimation of the errors of *D^U^* is only valid if its distribution can be approximated to a zero mean Gaussian distribution. Since such condition depends on several variables, a parameter to measure the validity of the estimation is necessary. For example, a second order Taylor series approximation could be used to evaluate higher order moments of the error distribution, such as the skewness and kurtosis [18]. In practice, however, we found that evaluating only the error perturbation on the least squares solution (Section 3.2) is enough to verify the reliability of the estimated variance. This is because a more restricted approximation is used for the pseudo-inverse linearization than for the other non-linear equations.

The first order Taylor series of the pseudo-inverse of matrix *A*′ = *A* + *Ã* can be expressed as [19]:

(20) $$\begin{array}{lll} {A^{\prime }}^{+} & \approx & A^{+}-A^{+}\;\tilde{A}A^{+}+{\left(A^{T}\;A\right)}^{-1}{\tilde{A}}^{T}\;P_{⊥}\\ & \approx & A^{+}+F \end{array}$$

where *P*_⊥_ = *I* − *AA*^+^ is the complementary orthogonal projection onto the orthogonal space of A. Here *F* represents the effect of noise over matrix *A*′. Since the approximation used in Equation (14) is valid when *A*^+^ ≫ *F*, a comparison between these terms is proposed in [16] by using a parameter *τ* that meets the condition:

(21) $$\tau \geq \frac{{\left\|F\right\|}_{S}}{\left\|A^{+}\right\|F}$$

where ||·||*_F_* represents the Frobenius norm and ||·||*_S_* the Stochastic norm as defined in [19]. The parameter *τ* represents the ratio between the error perturbation *F*, associated by the noisy measurements, and the real value of matrix *A*^+^. As the *τ* value increases, the perturbation became increasingly important and can not be disregarded.

In Appendix 7 it is shown that, assuming the general case where all elements of *A* and *B* can be dependent, *τ* can be expressed as:

(22) $$\begin{array}{c} \tau =\sqrt{\max\{m-1,2\times 5\}}\;{||Q^{\frac{1}{2}}\;A^{+}||}_{2} \end{array}$$

where the elements *q_uv_* of matrix *Q* ∈ ℝ^5×5^ are obtained from:

(23) $$q_{\mathrm{uv}}=\sum_{k=1}^{m-1}C({\tilde{a}}_{\mathrm{ku}},\;{\tilde{a}}_{\mathrm{kv}})$$

being *C* (*ã_ku_*, *ã_kv_*) the covariance between elements *u* and *v* of the *k*th row of matrix *Ã*. The covariance of these elements can be calculated by applying Equation (9) to the elements of matrix *A* defined in Equation (2).

## 4. Evaluation of the DSA Method

**Figure 2.**
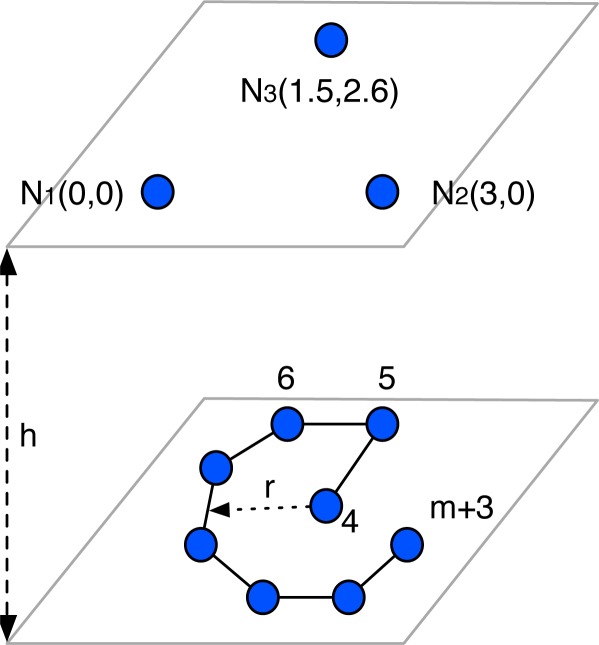
LPS configuration used for simulation composed by 3 beacons and a circular path with radius *r*, height *h* and *m* virtual nodes.

In order to evaluate the performance of the DSA method to predict the precision of the LAL inter-beacon solution, a LPS was simulated based on the node configuration shown in Figure 2. The measurement points are distributed along a circular path under the beacons, plus one measurement point at its center. In the following simulations, unless otherwise stated, the parameters are: radius *r* = 3 m, number of virtual nodes *m* = 12 and height *h* = 2*m*. The ranging data was generated with an additive white Gaussian noise with zero mean and a standard deviation of 0.01 m. All simulations were performed 1,000 times.

To avoid any confusion, we here define some terms used to refer to the different types of distance errors present in this section. We will refer as *input* errors those associated with the distance measurements *D^M^*. These errors have a known distribution (zero mean Gaussian noise) and a known standard deviation. The *output* errors are associated with the unknown inter-beacon distances *D^U^* obtained using the LAL algorithm. We call *simulated* values those obtained by simulating the auto-localization problem 1,000 times in order to calculate the output error statistics. In contrast the *estimated* values are those obtained using the methods and equations defined in this paper (Equations (18) and (22)). Finally *offline* estimates are those solutions obtained with the actual values of the distance measurements (*i.e.*, when we know the exact position of all nodes). The *online* estimates are solutions obtained with noisy distance measurements and the estimated position of the nodes obtained by the auto-localization method.

The offline estimation allows us to evaluate the limits of the DSA method under ideal conditions (*i.e.*, using the noise-free values of matrices *A* and *X* and the vector *B*). The estimation will be reliable as long as the output distribution of distances *D^U^* remains approximately Gaussian. As proposed in Section 3.4, the *τ* value should be sufficient to verify this reliability. The online method allows us to estimate “on-the-fly” the standard deviation of distances *D^U^* and use this information to improve the beacons’ localization. This method will present the same limits than the offline estimation, plus the effects of using the noisy matrices *A*′ and *X*′ and vector *B*′. The effect of the noisy matrices is evaluated by performing 1,000 times the online method on each test. The *τ* value will also be sufficient to verify the reliability of this method.

### 4.1. Standard Deviation Estimation Analysis

**Figure 3.**
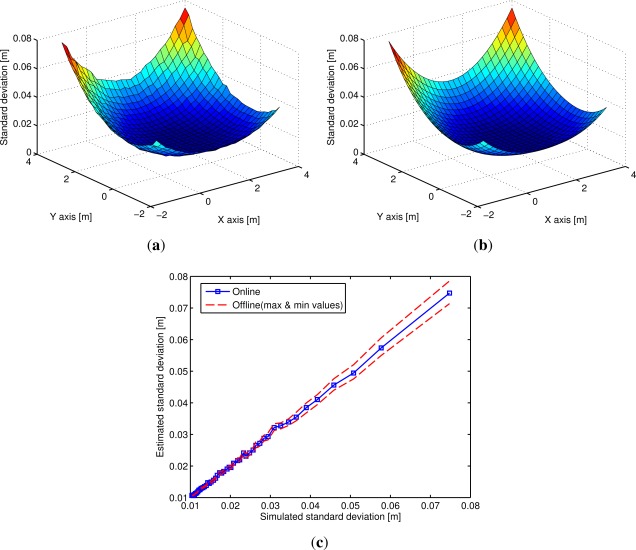
Output quadratic mean standard deviation *σ_mean_* obtained when changing the center of the virtual nodes path: (**a**) obtained by simulation (1,000 times); (**b**) estimated offline. In (**c**) estimated *σ_mean_* obtained with the offline and online methods compared to simulation.

To evaluate the DSA method we calculated the quadratic mean output standard deviation $\sigma_{\mathrm{mean}}=\frac{1}{\sqrt{3}}\sqrt{\sigma_{12}^{2}+\sigma_{13}^{2}+\sigma_{23}^{2}}$, of the inter-beacon distances *D^U^*, obtained when shifting the center of the virtual nodes path of the LPS configuration (Figure 2). The resultant standard deviation maps obtained by simulation (Figure 3(a)) and by offline estimation (Figure 3(b)) match almost exactly. The advantage of the offline estimation over the simulation is that the calculation processes of the maps are computationally more efficient. The evaluation of the standard deviation maps can be very useful to analyse the ideal route path of the virtual nodes. For example, in Figure 3(b) it is shown that the ideal position of the center of the path is near the central point of the beacons (1.5 m,1.3 m). If the center of the path is far from the central point, *σ_mean_* can increase up to 8 cm, that is, eight times the input standard deviation.

Figure 3(c) shows the estimated standard deviation obtained by the offline and online methods compared to the one obtained by simulation. As expected the online estimates’ errors are higher than the ones obtained by the offline method. Also, since the calculation of the vector *X* is directly related with the output standard deviation (Equation (16)), the online estimates worsens as the output standard deviation increases.

### 4.2. Reliability of the Variance Estimation

In Section 3.4 we proposed that the parameter *τ* can be used to weight the validity of the estimated output noise. To evaluate this premise we perform a simulation changing the two parameters that affect the output variance: the DDOP and the input variance. To change the DDOP we use the same LPS configuration as before but change the radius to the values *r* = {0.8 *m*, 1.2 *m*, 2 *m*} which produce a *ddop*_12_ = {7.79, 3.57, 1.52}. For the input variance a standard deviation ranging from 0 to 0.21 m is used.

**Figure 4.**
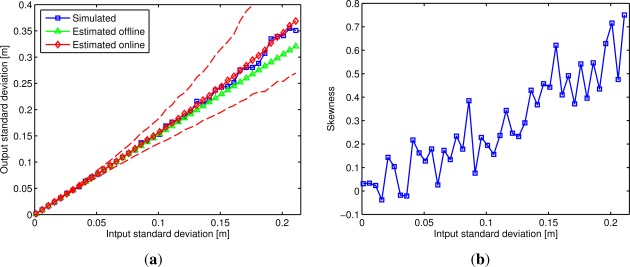
Evaluation of *d*_12_ when increasing the input standard deviation from 0 to 0.21 m. The used LPS configuration has a *ddop*_12_ of 1.52. In (**a**) the simulated, estimated offline and online output standard deviation. For the online estimates the mean and the 95% and 5% percentile are shown; In (**b**) the skewness of the distance *d*_12_ is shown.

In Figure 4(a) the simulated and estimated output standard deviation of distance *d*_12_ is shown with respect to the input standard deviation. For the online estimates the mean and the 95% and 5% percentile are shown. The graph corresponds to a *ddop*_12_ of 1.52. For an input noise less than 0.1 m, the output standard deviation increments almost linearly with the input standard deviation, as predicted by Equation (18). For higher values the simulation shows that the simulated standard deviation is always higher than the obtained from Equation (18). This happens because for high input noises the assumption that the error distribution of the output distances is close to a zero mean Gaussian distribution is no longer valid. This can be verified in Figure 4(b), where it is shown how the skewness of the distance *d*_12_ increases with the input noise. Since the skewness calculated for *d*_12_ is positive, the estimated standard deviation will be always lower than the one obtained in the simulation. This shows that the estimated offline standard deviation should not be used for a high input noise. For example, a 0.1 m input noise limit can be chose for this particular LPS configuration. Figure 4(a) also shows, as expected, that the estimated online standard deviation presents a higher error with the increment of the input noise.

**Figure 5.**
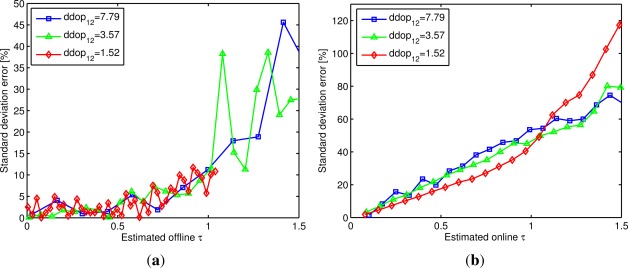
Calculated standard deviation errors of distance *d*_12_ using a LPS configuration with *ddop*_12_ = {7.79, 3.57, 1.52} and a standard deviation input error ranging from 0 to 0.21 m. The correlation between the value *τ* and the estimated standard deviation error is shown for offline estimation in (**a**) and for online estimates (the 95% percentile) in (**b**).

Figure 5 shows the offline and 95% percentile online standard deviation error in percentage (compared to the one obtained by simulation) as a function of the *τ* value. These results are obtained for a *ddop*_12_ = {7.79, 3.57, 1.52} and a standard deviation input error ranging from 0 to 0.21 m. The graphics are limited to values of 0 ≤ *τ* ≤ 1.5 to focus within a reasonable range, since for values of *τ* > 1.5 the errors are much higher than the ones shown in the graphics.

Figure 5(a) shows the standard deviation error in percentage obtained with the offline method. This error is always under 10% for *τ* values inferior to 0.7, and within this range it seems almost constant regardless of the value of *τ*. For *τ >* 0.7 the error increases with *τ* . It is clear that, at this point, the effects of the skewness of the output distances begin to be appreciable. Notice that the value of *τ* where the standard deviation error begins to increase seems to be independent of the *ddop*_12_ value. This shows that the *τ* value is sufficient to verify the reliability of Equation (18) for the offline method, regardless of the input errors or the geometry of the LPS system.

A similar analysis used for the offline estimation can be used for the online case. Figure 5(b) shows the 95% percentile of the standard deviation error obtained with the online method in percentage. Since for every of the 1,000 iterations a slightly different value of *τ* is obtained (due to noisy matrices) we grouped *τ* in fixed intervals of 0.05 values (e.g., any value of *τ* between 1 and 1.05 is represented in the graph as a value of *τ* = 1). It can be seen that for *τ* < 0.7 the standard deviation error is always under 40%, though within this range the error is not constant and always increases with the value of *τ* . This effect is generated by the noise perturbation over the matrices *A*′ and *B*′. Still, we can use *τ* to limit the maximum error obtained when estimating the output standard deviation. For example, to obtain errors not higher than 20% a value of *τ* ≤ 0.3 can be chosen.

In this case, the value *τ* where the standard deviation error is lower than a given limit is not totally independent of the *dop*_12_ value, so it is possible that for higher values of DDOP or input noise the error could be higher. However, in practical applications, the values of the DDOP, the input noise, and *τ* will mostly be lower than the ones here simulated. For example, the value of *τ* obtained in the tests performed in Section 4.1 was always of *τ* ≤ 0.13

## 5. Weighted Linearized Auto-Localization Algorithm (WLAL)

In this section a new algorithm of auto-localization is presented based on the LAL algorithm and the error estimation techniques exposed in Section 3. As presented in [10], and reviewed in Section 2, the LAL algorithm calculates the distances between the unlocated beacons that are later used to obtain their relative position. The algorithm calculates the inter-beacon distances of every subset of three beacons, therefore more than one distance estimation is usually obtained between any pair of beacons. The final distance between two beacons is obtained by calculating the mean of all the available distances. We propose to rather use a weighted mean based on the online estimated standard deviations of the distances using the method presented in Section 3. If for each inter-beacon distance *d_ij_* we obtain *k* distances estimations, the respective weighted mean distance *d̄_ij_* is obtained by:

(24) $${\bar{d}}_{\mathrm{ij}}=\frac{\sum_{k=1}^{n}(d_{\mathrm{ij}})k/{\left(\sigma_{\mathrm{ij}}^{2}\right)}_{k}}{\sum_{k=1}^{n}1/{\left(\sigma_{\mathrm{ij}}^{2}\right)}_{k}}$$

where (*d_ij_*)*_k_* is the *k*-th distance *d_ij_* estimation and (*σ_ij_*)*_k_* the respective standard deviation that can be calculated from Equation (19).

By using the estimated standard deviation for each possible subset, we can give more weight to solutions with the best subset of beacons. With the WLAL algorithm we expect an improvement of the resultant standard deviation of the inter-beacon distances. The expected variance *σ̄_ij_* for each interbeacon distance *d̄_ij_* obtained by applying the weighted mean is:

(25) $${\bar{\sigma }}_{\mathrm{ij}}^{2}=\frac{1}{\sum_{k=1}^{n}1/{\left(\sigma_{\mathrm{ij}}^{2}\right)}_{k}}$$

## 6. Evaluation on an Ultrasonic LPS

**Figure 6.**
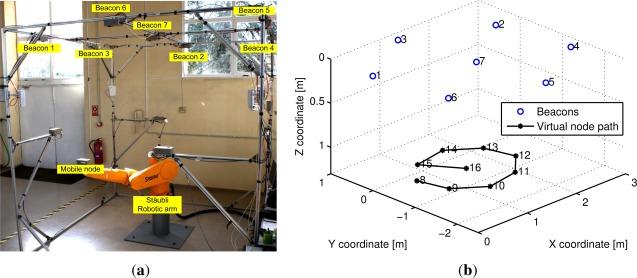
3DLocus beacons and virtual nodes configuration used in the test. (**a**) 3DLocus acoustic localization system; (**b**) Nodes configuration used in the test.

We apply the DSA method on a real ultrasonic LPS, where the assumptions of co-planarity between beacons and the presence of only Gaussian noise in the measurements are not completely true. We want to evaluate the performance of the method under non-ideal conditions. The test is performed on the 3DLocus system [4], shown in Figure 6(a), which is an acoustic LPS composed by n = 7 beacons deployed on a cell of 2.8 m × 2.8 m × 2.8 m. The calculated standard deviation of the distance measurements obtained with the 3DLocus is 0.23 mm, which is much more accurate than the one used in the simulations in Section 4. The height between the beacons and the virtual nodes is 1.4 m.

The linearized auto-localization method is compared with the inverse positioning method presented in [2]. Since the inverse positioning method requires the exact position of the mobile node, a Staübli Unimation industrial robotic arm with a 50 *μ*m accuracy was used for positioning such node. An approximately circular path with a radius of 0.8 m of m = 9 virtual nodes were used in this test, and a total of 100 measurements were made on each point. From these measurements we obtain 100 individual estimations of the positions of the beacons. The nodes’ configuration used in this test is shown in Figure 6(b).

### 6.1. Inter-Beacon Distances Estimation

**Figure 7.**
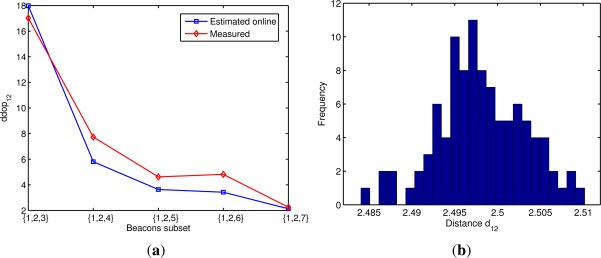
Experimental evaluation of the estimated distance *d*_12_ obtained with different beacons’ subsets. In (**a**) the estimated online and measured *ddop*_12_; In (**b**) the histogram of the calculated distance *d*_12_ obtained using the beacons’ subset {1, 2, 3}.

Figure 7(a) shows the estimated online and measured distance dilution of precision *ddop*_12_ obtained with different subsets of beacons. The measured *ddop*_12_ was calculated by dividing the standard deviation of the calculated distance *d*_12_ by the standard deviation of the distance measurements *D^M^*. In most cases the estimated value is slightly lower than the actual *ddop*_12_ obtained in the 3DLocus, though the difference is always below 30%. One reason for this difference is that we are using a mean standard deviation of 0.23 mm for all the 3DLocus measurements while the actual value of the measurements’ errors depends on various factors such as the distance and angle between nodes. In Figure 7(b) the histogram of the calculated distance *d*_12_ obtained using the beacons’ subset {1, 2, 3} is shown. As can be observed, the error distribution of the calculated distance resembles a Gaussian distribution. In order to verify the assumption of a Gaussian error distribution on all the calculated inter-beacon distances, we run a Lilliefors test for normality [20]. The test established with a *p*-value of 0.05 that the error distribution on the calculated inter-beacon distances approximates a normal distribution (*i.e.*, there is a 5% probability that the normality is a false positive).

In Table 1 a comparison of the output standard deviation *σ_l_* obtained with the LAL algorithm and the standard deviation *σ_w_* obtained with the WLAL algorithm are shown. In every case *σ_w_* was lower than *σ_l_*. For example, for the distance *d*_12_ the standard deviation obtained by the WLAL algorithm is 65% less than the one obtained with the LAL algorithm. Since we know that the ideal position of the virtual nodes path is under the center of the triangle formed by the subset of three beacons, it is clear that the solution obtained with the subset {1, 2, 3} will have a higher error than the one obtained with the subset {1, 2, 5}, when estimating the *d*_12_ distance. On average, an improvement of 32.8% was obtained using the WLAL algorithm.

### 6.2. Beacons’ Position Estimation

**Figure 8.**
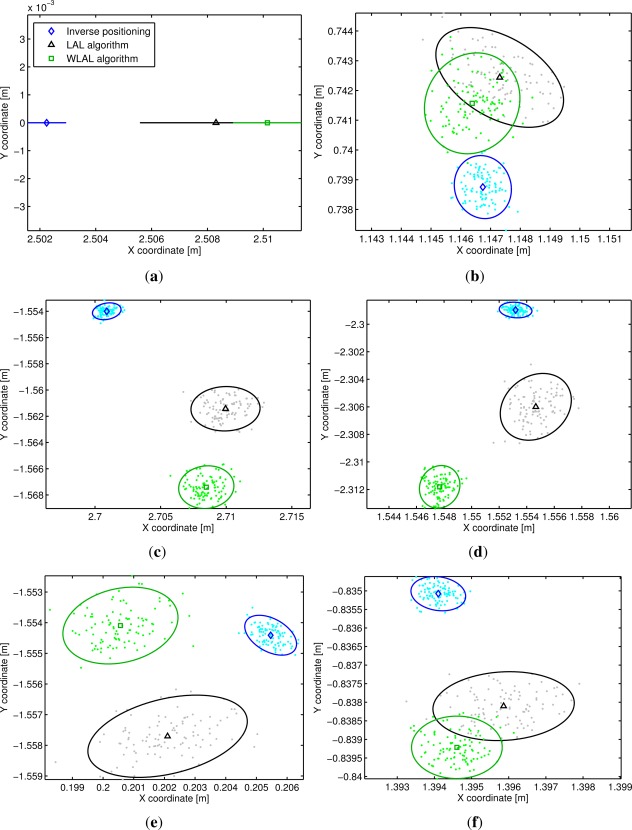
X-Y coordinates of 3DLocus beacons’ positions obtained with the LAL algorithm, the WLAL algorithm and the inverse positioning method. The solid lines represent the 90% confidence ellipses of the estimated positions. (**a**) Beacon 2; (**b**) Beacon 3; (**c**) Beacon 4; (**d**) Beacon 5; (**e**) Beacon 6; (**f**) Beacon 7.

In Figure 8 the beacons’ position estimation obtained over one hundred trials with the LAL method, WLAL algorithm and the inverse positioning method is shown on the X-Y plane. A coordinate system was defined using the beacon 1 as origin and the beacon 2 as the X axis. As expected the standard deviation of the beacons’ position is improved by using the weighted mean instead of a simple mean of the estimated inter-beacon distances. On average, an improvement of 22% was obtained on the standard deviation on axis X and Y using the WLAL algorithm.

Figure 8 also shows bias errors between the auto-localization methods. Using the inverse positioning solution as the ground truth location of the beacons, a mean RMS error of the beacons’ positions of 0.76 cm and 0.92 cm is obtained with the LAL and WLAL algorithms respectively. The greatest bias observed is for the fifth beacon (1.3 cm). The presence of a bias between the different methods could be originated by several causes. First, although the cell structure of the 3DLocus is positioned parallel to the floor, it seems that there is a small inclination that causes a height variation on the beacons. The inverse positioning method shows a mean height variation of up to 8 mm between the beacons. Since the LAL and WLAL methods assume that the beacons are on a plane, the height difference will generate a discrepancy between the inverse positioning and the linearized methods. A second source of the observed bias could be the noisy distances used in the solution of the inter-beacon distances. In this paper we are only taking into account the influence of the noise using a first order approximation, however, it is known that the higher terms of the noise can also generate a bias [21].

**Figure 9.**
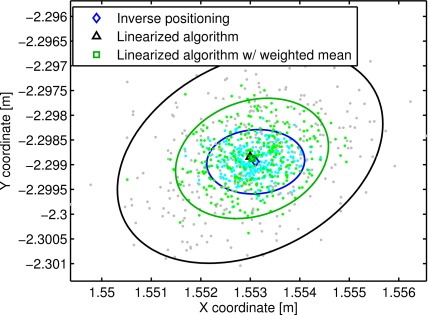
X-Y coordinates of beacon 5 obtained by simulation. The simulation does not presents the bias observed in the experimental test with the 3Dlocus LPS.

To evaluate these hypotheses, we simulated a LPS using the positions obtained with the inverse positioning as the real location of the beacons, but positioning them in the same plane (by setting the *Z* coordinate to be zero on all beacons) and using simulated measurements with the same standard deviation. The simulation showed no bias between the methods in any beacon, as can be seen for the fifth beacon in Figure 9, showing that the effect of the higher terms of the noise is negligible. When the beacons were not placed in the same plane, the simulation showed a bias between all the auto-localization methods. Using the beacon’s altitude obtained with the inverse positioning, the simulation showed a bias of 5 mm for the fifth beacon. Finally, systematic range errors originated from the actual LPS can also add to the bias observed [22] (e.g., the range measurement is affected by the orientation between the beacon and the mobile node). Any error originated by this bias and also the small height variation between beacons can be compensated using an optimization algorithm such as the ones used in [6–8]. The WLAL solution could be used as a first estimation of the beacons position, since with a 1.5 cm offset any optimization algorithm will easily converge to a more accurate solution.

## 7. Conclusions

In this paper a method to estimate the error of the solutions obtained with the Linearized Auto-Localization (LAL) method was presented. The method is based on the differential sensitivity analysis that uses a first order Taylor approximation to obtain the function’s error variance. Since the method depends on such approximation, a confidence parameter *τ* was defined to measure the reliability of the estimated error distribution. The differential sensitivity analysis showed that the standard deviation of the solution, obtained by the LAL method, is proportional to the standard deviation of the distance measurements and a matrix*M_DOP_* dependent of the geometry of the localization system, which is similar to the geometric dilution of precision on localization problems.

Two versions of the error estimation were evaluated: (a) the offline method that requires the location of all nodes and (b) the online method where all the nodes’ positions are unknown. The first method is useful to evaluate possible path routes and strategies that maximize the accuracy of the auto-localization algorithm. The advantage of this method is that the calculation processes are computationally more efficient than an evaluation based on a simulation process. Simulated test showed that *τ* can be used to limit the maximum error obtained by the offline method regardless of the measured distances noise and the DDOP. The online method can be used to estimate “on-the-fly” the standard deviation of the inter-beacon distances obtained by the auto-localization algorithm, and use this information to improve its accuracy. It was also shown by simulation that the parameter *τ* can be used to limit the maximum error obtained with the online method.

Finally, a modification of the LAL method was used to evaluate the online method on an acoustic LPS. For the calculation of the inter-beacons distances, a weighted mean rather than a simple mean was proposed by using the output error statistics obtained by the online method. By using this Weighted Linearized Auto-Localization (WLAL) algorithm, the standard deviation of the calculated inter-beacon distances showed an improvement of 32.8% on average. The beacons’ position estimation also presented a improvement of 22% of the standard deviation on axes X and Y.

## Figures and Tables

**Table 1.** Standard deviation of the inter-beacon distances *d_ij_* obtained using the mean *σ_l_* and the weighted mean *σ_w_*.

***d_ij_***	***σ_l_*[*cm*]**	***σ_w_*[*cm*]**	***d_ij_***	***σ_l_*[*cm*]**	***σ_w_*[*cm*]**	***d_ij_***	***σ_l_*[*cm*]**	***σ_w_*[*cm*]**
*d*_12_	0.118	0.041	*d*_24_	0.085	0.058	*d*_37_	0.060	0.047
*d*_13_	0.091	0.064	*d*_25_	0.094	0.046	*d*_45_	0.054	0.041
*d*_14_	0.115	0.103	*d*_26_	0.104	0.102	*d*_46_	0.074	0.030
*d*_15_	0.090	0.036	*d*_27_	0.058	0.041	*d*_47_	0.048	0.029
*d*_16_	0.071	0.049	*d*_34_	0.116	0.053	*d*_56_	0.060	0.047
*d*_17_	0.053	0.043	*d*_35_	0.165	0.144	*d*_57_	0.044	0.033
*d*_23_	0.076	0.055	*d*_36_	0.099	0.036	*d*_67_	0.046	0.040

## Appendix—Calculation of the *τ* Value

To obtain the value of *τ* we begin with the first order expansion of *F* derived from Equation (20):

(26) $$F=-A^{+}\tilde{A}A^{+}+{\left(A^{T}\;A\right)}^{-1}{\tilde{A}}^{T}\;P_{⊥}$$

Let ||*F*||*_S_* be the stochastic norm defined as:

(27) $${\left\|F\right\|}_{S}=\sqrt{𝔼({||F||}_{F}^{2})}$$

where 𝔼 represents the expected value. The correspondent squared stochastic norm of *F* using Equation (26) is:

(28) $${||F||}_{S}^{2}={||-A^{+}\tilde{A}A^{+}+{\left(A^{T}\;A\right)}^{-1}{\tilde{A}}^{T}\;P_{⊥}||}_{S}^{2}$$

which based on [19] (Section 3.1.3) can be written as:

(29) $${||F||}_{S}^{2}={||-A^{+}\tilde{A}A^{+}||}_{S}^{2}+{||{\left(A^{T}\;A\right)}^{-1}{\tilde{A}}^{T}\;P_{⊥}||}_{S}^{2}$$

Beginning with the first term of Equation (29) we have:

(30) $$\begin{array}{lll} {||-A^{+}\tilde{A}\;A^{+}||}_{S}^{2} & = & 𝔼\;\left({||-A^{+}\tilde{A}\;A^{+}||}_{F}^{2}\right)\\ & \leq & 𝔼\;\left({||A^{+}||}_{F}^{2}\;{||-\tilde{A}\;A^{+}||}_{F}^{2}\right)\\ & \leq & {||A^{+}||}_{F}^{2}\;𝔼\;\left({||-\tilde{A}\;A^{+}||}_{F}^{2}\right) \end{array}$$

The Frobenius norm of the second term in Equation (30) can be simplified as:

(31) $$\begin{array}{lll} 𝔼\;\left({||-\tilde{A}\;A^{+}||}_{F}^{2}\right) & = & 𝔼\;\left(\mathrm{trace}\;\left({\left(\tilde{A}\;A^{+}\right)}^{T}\;\tilde{A}\;A^{+}\right)\right)\\ & = & 𝔼\;\left(\mathrm{trace}\left(A^{+T}\;{\tilde{A}}^{T}\;\tilde{A}\;A^{+}\right)\right)\\ & = & \mathrm{trace}\;\left(A^{+T}\;𝔼\left({\tilde{A}}^{T}\;\tilde{A}\right)\;A^{+}\right)\\ & = & \mathrm{trace}\;\left(A^{+T}\;QA^{+}\right) \end{array}$$

where the elements *q_uv_* of matrix *Q* ∈ ℝ^5×5^ can be calculated by:

(32) $$\begin{array}{lll} q_{\mathrm{uv}} & = & 𝔼\;\left(\mathrm{row}\;{\left({\tilde{A}}^{T}\right)}_{u}\times \mathrm{column}\;{\left(\tilde{A}\right)}_{v}\right)\\ & = & 𝔼\;\left(\mathrm{column}\;{\left(\tilde{A}\right)}_{u}^{T}\times \mathrm{column}\;{\left(\tilde{A}\right)}_{v}\right)\\ & = & 𝔼\;\left({\tilde{a}}_{1u}\times {\tilde{a}}_{1v}+{\tilde{a}}_{2u}\times {\tilde{a}}_{2v}+\cdots +{\tilde{a}}_{(m-1)u}\times {\tilde{a}}_{(m-1)v}\right) \end{array}$$

Since we assume that all elements of the random matrix *Ã* have mean zero, we have that:

(33) $$𝔼\;({\tilde{a}}_{\mathrm{ku}}\times {\tilde{a}}_{\mathrm{kv}})=C({\tilde{a}}_{\mathrm{ku}},\;{\tilde{a}}_{\mathrm{kv}})$$

therefore Equation (32) can be simplified as:

(34) $$q_{\mathrm{uv}}=\sum_{k=1}^{\left(m-1\right)}C({\tilde{a}}_{\mathrm{ku}},\;{\tilde{a}}_{\mathrm{kv}})$$

Since matrix *Q* is symmetric and also positive semidefinite (since it is the product of (*Ã^T^Ã*)), we can define *Q*^1/2^ as the symmetric square root of *Q* where:

(35) $${\left(Q^{1/2}\right)}^{T}\;Q^{1/2}=Q$$

Replacing Equation (35) in Equation (31) we obtain:

(36) $$\begin{array}{lll} 𝔼\left({||-\tilde{A}\;A^{+}||}_{F}^{2}\right) & = & \mathrm{trace}\;(A^{+T}\;{\left(Q^{1/2}\right)}^{T}\;Q^{1/2}\;A^{+})\\ & = & {||Q^{1/2}\;A^{+}||}_{F}^{2} \end{array}$$

So the first term of the squared stochastic norm of *F* Equation (29) can be written as:

(37) $${||-A^{+}\;\tilde{A}\;A^{+}||}_{S}^{2}\leq {||A^{+}||}_{F}^{2}\;{||Q^{1/2}\;A^{+}||}_{F}^{2}$$

A similar procedure can be used to simplify the second term of Equation (29), obtaining:

(38) $${||{\left(A^{T}\;A\right)}^{-1}\;{\tilde{A}}^{T}\;P_{⊥}||}_{S}^{2}\leq {||Q^{1/2}{\left(A^{T}\;A\right)}^{-1}||}_{F}^{2}\;{||P_{⊥}||}_{F}^{2}$$

Hence:

(39) $${||F||}_{S}^{2}\leq {||A^{+}||}_{F}^{2}\;{||Q^{1/2}\;A^{+}||}_{F}^{2}+{||Q^{1/2}\;{\left(A^{T}\;A\right)}^{-1}||}_{F}^{2}\;{||P_{⊥}||}_{F}^{2}$$

Based on [16] (Section 7), from Equation (39) we obtain the expression of *τ* as:

(40) $$\frac{{||F||}_{S}}{{||A^{+}||}_{F}}\leq \sqrt{\max\{m-1,2\times 5\}}\;{||Q^{\frac{1}{2}}\;A^{+}||}_{2}\equiv \tau$$

since *A* ∈ ℝ^(*m*−1)×5^. From which we obtain Equation (22).
